# Supplementary material for: Mutations in filamentous bacteriophages spark eco-evolutionary feedbacks in Pseudomonas aeruginosa
Source: ISME J. 2026 Aug 4;20(1):wrag193. doi: 10.1093/ismejo/wrag193 (PMC13502202; doi:10.1093/ismejo/wrag193)
Supplement: Supplementary file 1 [file web_material_wrag193.zip › NCEE - Prophage Paper - Supplementary Table 1.pdf]

Supplementary Table 1: Genetic variants detected in hyperactive filamentous phage isolates.

Phage Isolate ID	Phage Species	Gene Name	Gene Product	Type	SNP Type	Genetic Change	Amino Acid Change
HD1_0500_phi1	Pf6	MPAO1_RS28990/MPAO1_RS24230	transcriptional regulator/hypothetical protein	DEL		Δ10bp	N/A
HD1_0500_phi1	Pf6	MPAO1_RS28990/MPAO1_RS24230	transcriptional regulator/hypothetical protein	SNP	intergenic	C→T	N/A
HD1_0500_phi1	Pf6	MPAO1_RS24215	hypothetical protein	SNP	nonsynonymous	A→G	E18G
HD1_0750_phi1	Pf6	MPAO1_RS28990/MPAO1_RS24230	transcriptional regulator/hypothetical protein	DEL		Δ10bp	N/A
HD1_0750_phi1	Pf6	MPAO1_RS28990/MPAO1_RS24230	transcriptional regulator/hypothetical protein	SNP	intergenic	C→T	N/A
HD1_0750_phi1	Pf6	MPAO1_RS24215	hypothetical protein	SNP	nonsynonymous	A→C	Q8P
HD1_0750_phi2	Pf6	MPAO1_RS28990/MPAO1_RS24230	transcriptional regulator/hypothetical protein	DEL		Δ10bp	N/A
HD1_0750_phi2	Pf6	MPAO1_RS28990/MPAO1_RS24230	transcriptional regulator/hypothetical protein	SNP	intergenic	C→T	N/A
HD1_0750_phi2	Pf6	MPAO1_RS24215	hypothetical protein	SNP	nonsynonymous	A→C	Q8P
HD7_0250_phi1	Pf4	PA0714.1/PA0715	PhrD/polymerase	SNP	intergenic	G→C	N/A
HD7_0250_phi1	Pf4	[PA0715]–PA0716.1	[PA0715],PA0716,PA0716.1	DEL		Δ2859bp	N/A
HD7_0250_phi1	Pf4	PA0722	hypothetical protein	SNP	nonsynonymous	A→C	Q8P
HD7_0250_phi2	Pf4	PA0714.1/PA0715	PhrD/polymerase	SNP	intergenic	G→C	N/A
HD7_0250_phi2	Pf4	PA0722	hypothetical protein	SNP	nonsynonymous	A→C	Q8P
HD7_0250_phi3	Pf4	PA0714.1/PA0715	PhrD/polymerase	SNP	intergenic	G→C	N/A
HD7_0250_phi3	Pf4	[PA0715]–PA0716.1	[PA0715],PA0716,PA0716.1	DEL		Δ2859bp	N/A
HD7_0250_phi3	Pf4	PA0722	hypothetical protein	SNP	nonsynonymous	A→C	Q8P
HD7_0250_phi3	Pf4	PA0726	CTXphi Zot-like toxin	SNP	nonsynonymous	C→T	T41I
HD7_0500_phi1	Pf4	PA0714.1/PA0715	PhrD/polymerase	SNP	intergenic	G→C	N/A
HD7_0500_phi1	Pf4	PA0722	hypothetical protein	SNP	nonsynonymous	A→C	Q8P
HD7_0500_phi1	Pf4	PA0724	hypothetical protein	SNP	nonsynonymous	G→A	R53H
HD7_0500_phi1	Pf4	PA0724	hypothetical protein	SNP	nonsynonymous	A→G	D95G
HD7_0500_phi1	Pf4	PA0726	CTXphi Zot-like toxin	SNP	nonsynonymous	C→T	R355W
HD7_0500_phi2	Pf4	PA0714.1/PA0715	PhrD/polymerase	SNP	intergenic	G→C	N/A
HD7_0500_phi2	Pf4	PA0722	hypothetical protein	SNP	nonsynonymous	A→C	Q8P
HD7_0500_phi2	Pf4	PA0724	hypothetical protein	SNP	nonsynonymous	G→A	R53H
HD7_0500_phi2	Pf4	PA0724	hypothetical protein	SNP	nonsynonymous	A→G	D95G
HD7_0500_phi2	Pf4	PA0726	CTXphi Zot-like toxin	SNP	nonsynonymous	C→T	R355W
HD7_0500_phi3	Pf4	PA0714.1/PA0715	PhrD/polymerase	SNP	intergenic	G→C	N/A
HD7_0500_phi3	Pf4	PA0722	hypothetical protein	SNP	nonsynonymous	A→C	Q8P
HD7_0500_phi3	Pf4	PA0724	hypothetical protein	SNP	nonsynonymous	G→A	R53H
HD7_0500_phi3	Pf4	PA0724	hypothetical protein	SNP	nonsynonymous	A→G	D95G
HD7_0500_phi3	Pf4	PA0726	CTXphi Zot-like toxin	SNP	nonsynonymous	C→T	R355W
HD7_0750_phi1	Pf4	PA0714.1/PA0715	PhrD/polymerase	SNP	intergenic	G→C	N/A
HD7_0750_phi1	Pf4	[PA0715]–PA0716.1	[PA0715],PA0716,PA0716.1	DEL		Δ2859bp	N/A

HD7_0750_phi1	Pf4	PA0726	CTXphi Zot-like toxin	SNP	nonsynonymous	C→T	R355W
HD7_0750_phi1	Pf4	PA0729	hypothetical protein	SNP	nonsynonymous	C→T	P3L
HD7_0750_phi2	Pf4	PA0714.1/PA0715	PhrD/polymerase	SNP	intergenic	G→C	N/A
HD7_0750_phi2	Pf4	PA0724	hypothetical protein	SNP	nonsynonymous	G→A	R53H
HD7_0750_phi2	Pf4	PA0724	hypothetical protein	SNP	nonsynonymous	A→G	D95G
HD7_0750_phi2	Pf4	PA0726	CTXphi Zot-like toxin	SNP	nonsynonymous	C→T	R355W
HD7_0750_phi3	Pf4	PA0714.1/PA0715	PhrD/polymerase	SNP	intergenic	G→C	N/A
HD7_0750_phi3	Pf4	PA0720	single strand DNA binding protein	SNP	nonsynonymous	C→T	P82S
HD7_0750_phi3	Pf4	PA0722	hypothetical protein	SNP	nonsynonymous	A→C	Q8P
HD7_0750_phi3	Pf4	PA0722	hypothetical protein	SNP	nonsynonymous	G→T	A27S
HD7_0750_phi3	Pf4	PA0724	hypothetical protein	SNP	nonsynonymous	G→A	R53H
HD7_0750_phi3	Pf4	PA0724	hypothetical protein	SNP	nonsynonymous	A→G	D95G
HD7_0750_phi3	Pf4	PA0726	CTXphi Zot-like toxin	SNP	nonsynonymous	C→T	R355W
HD7_1000_phi1	Pf4	PA0714.1/PA0715	PhrD/polymerase	SNP	intergenic	G→C	N/A
HD7_1000_phi1	Pf4	PA0720	single strand DNA binding protein	SNP	nonsynonymous	C→T	P82S
HD7_1000_phi1	Pf4	PA0722	hypothetical protein	SNP	nonsynonymous	A→C	Q8P
HD7_1000_phi1	Pf4	PA0722	hypothetical protein	SNP	nonsynonymous	G→T	A27S
HD7_1000_phi1	Pf4	PA0724	hypothetical protein	SNP	nonsynonymous	G→A	R53H
HD7_1000_phi1	Pf4	PA0724	hypothetical protein	SNP	nonsynonymous	A→G	D95G
HD7_1000_phi1	Pf4	PA0726	CTXphi Zot-like toxin	SNP	nonsynonymous	C→T	R355W
HD7_1000_phi2	Pf4	PA0714.1/PA0715	PhrD/polymerase	SNP	intergenic	G→C	N/A
HD7_1000_phi2	Pf4	[PA0715]–PA0716.1	[PA0715],PA0716,PA0716.1	DEL		Δ2859bp	N/A
HD7_1000_phi2	Pf4	PA0720	single strand DNA binding protein	SNP	nonsynonymous	C→T	P82S
HD7_1000_phi2	Pf4	PA0722	hypothetical protein	SNP	nonsynonymous	A→C	Q8P
HD7_1000_phi2	Pf4	PA0722	hypothetical protein	SNP	nonsynonymous	G→T	A27S
HD7_1000_phi2	Pf4	PA0724	hypothetical protein	SNP	nonsynonymous	G→A	R53H
HD7_1000_phi2	Pf4	PA0724	hypothetical protein	SNP	nonsynonymous	A→G	D95G
HD7_1000_phi2	Pf4	PA0726	CTXphi Zot-like toxin	SNP	nonsynonymous	C→T	R355W
HD7_1000_phi3	Pf4	PA0714.1/PA0715	PhrD/polymerase	SNP	intergenic	G→C	N/A
HD7_1000_phi3	Pf4	[PA0715]–PA0716.1	[PA0715],PA0716,PA0716.1	DEL		Δ2859bp	N/A
HD7_1000_phi3	Pf4	PA0720	single strand DNA binding protein	SNP	nonsynonymous	C→T	P82S
HD7_1000_phi3	Pf4	PA0722	hypothetical protein	SNP	nonsynonymous	A→C	Q8P
HD7_1000_phi3	Pf4	PA0722	hypothetical protein	SNP	nonsynonymous	G→T	A27S
HD7_1000_phi3	Pf4	PA0724	hypothetical protein	SNP	nonsynonymous	G→A	R53H
HD7_1000_phi3	Pf4	PA0724	hypothetical protein	SNP	nonsynonymous	A→G	D95G
HD7_1000_phi3	Pf4	PA0726	CTXphi Zot-like toxin	SNP	nonsynonymous	C→T	R355W
LD6_0250_phi1	Pf6*	MPAO1_RS28990/MPAO1_RS24230	transcriptional regulator/hypothetical protein	SNP	intergenic	C→T	N/A
LD6_0250_phi1	Pf6*	MPAO1_RS28990/MPAO1_RS24230	transcriptional regulator/hypothetical protein	DEL		Δ4bp	N/A
LD6_0250_phi1	Pf6*	MPAO1_RS24225	hypothetical protein	SNP	nonsynonymous	C→T	A27V
LD6_0250_phi1	Pf6*	MPAO1_RS24225	hypothetical protein	SNP	synonymous	G→A	P37P
LD6_0250_phi1	Pf6*	MPAO1_RS24225	hypothetical protein	SNP	synonymous	T→C	D38D
LD6_0250_phi1	Pf6*	MPAO1_RS24225	hypothetical protein	SNP	synonymous	C→T	C39C
LD6_0250_phi1	Pf6*	MPAO1_RS24225	hypothetical protein	SNP	nonsynonymous	A→G	D49G

LD6_0250_phi1	Pf6*	MPAO1_RS24225	hypothetical protein	SNP	synonymous	A→G	K81K
LD6_0250_phi1	Pf6*	MPAO1_RS24225	hypothetical protein	SNP	synonymous	T→C	P82P
LD6_0250_phi1	Pf6*	MPAO1_RS24225	hypothetical protein	SNP	synonymous	C→T	F85F
LD6_0250_phi1	Pf6*	MPAO1_RS24225/ZDSLLIWT_CDS_0006	hypothetical protein/hypothetical protein	SNP	intergenic	C→A	N/A
LD6_0250_phi1	Pf6*	ZDSLLIWT_CDS_0006	hypothetical protein	SNP	synonymous	C→T	L2L
LD6_0250_phi1	Pf6*	ZDSLLIWT_CDS_0006	hypothetical protein	SNP	nonsynonymous	T→C	L36P
LD6_0250_phi1	Pf6*	ZDSLLIWT_CDS_0006	hypothetical protein	SNP	synonymous	C→G	R46R
LD6_0250_phi1	Pf6*	ZDSLLIWT_CDS_0006	hypothetical protein	SNP	synonymous	T→G	L60L
LD6_0250_phi1	Pf6*	ZDSLLIWT_CDS_0006	hypothetical protein	INS			N/A
LD6_0250_phi1	Pf6*	ZDSLLIWT_CDS_0006	hypothetical protein	SNP	synonymous	C→A	G102G
LD6_0250_phi1	Pf6*	ZDSLLIWT_CDS_0006	hypothetical protein	SNP	nonsynonymous	C→T	T109I
LD6_0250_phi1	Pf6*	ZDSLLIWT_CDS_0006	hypothetical protein	SNP	nonsynonymous	A→C	T111P
LD6_0250_phi1	Pf6*	ZDSLLIWT_CDS_0006	hypothetical protein	SNP	synonymous	T→C	T111P
LD6_0250_phi1	Pf6*	ZDSLLIWT_CDS_0006	hypothetical protein	SNP	nonsynonymous	T→C	*115R
LD6_0250_phi1	Pf6*	MPAO1_RS24220	single strand DNA binding protein	SNP	synonymous	T→C	T18T
LD6_0250_phi1	Pf6*	MPAO1_RS24220	single strand DNA binding protein	SNP	synonymous	C→T	Y21Y
LD6_0250_phi1	Pf6*	MPAO1_RS24220	single strand DNA binding protein	SNP	synonymous	G→A	Q34Q
LD6_0250_phi1	Pf6*	MPAO1_RS24220	single strand DNA binding protein	SNP	synonymous	C→T	G42G
LD6_0250_phi1	Pf6*	MPAO1_RS24220	single strand DNA binding protein	SNP	synonymous	C→A	G49G
LD6_0250_phi1	Pf6*	MPAO1_RS24220	single strand DNA binding protein	SNP	synonymous	A→G	P71P
LD6_0250_phi1	Pf6*	MPAO1_RS24220	single strand DNA binding protein	SNP	synonymous	C→T	R81R
LD6_0250_phi1	Pf6*	MPAO1_RS24220	single strand DNA binding protein	SNP	synonymous	T→C	G88G
LD6_0250_phi1	Pf6*	MPAO1_RS24220	single strand DNA binding protein	SNP	synonymous	A→G	Q94Q
LD6_0250_phi1	Pf6*	MPAO1_RS24220	single strand DNA binding protein	SNP	synonymous	T→A	L96L
LD6_0250_phi1	Pf6*	MPAO1_RS24220	single strand DNA binding protein	SNP	synonymous	T→C	G104G
LD6_0250_phi1	Pf6*	MPAO1_RS24220	single strand DNA binding protein	SUB		Δ2bp	N/A
LD6_0250_phi1	Pf6*	MPAO1_RS24220	single strand DNA binding protein	SUB		Δ2bp	N/A
LD6_0250_phi1	Pf6*	MPAO1_RS24220	single strand DNA binding protein	SNP	nonsynonymous	T→A	S139T
LD6_0250_phi1	Pf6*	MPAO1_RS24220	single strand DNA binding protein	SNP	synonymous	C→G	A144A
LD6_0250_phi1	Pf6*	MPAO1_RS24220/ZDSLLIWT_CDS_0008	single strand DNA binding protein/hypothetical protein	SNP	intergenic	G→C	N/A
LD6_0250_phi1	Pf6*	ZDSLLIWT_CDS_0008	hypothetical protein	SNP	synonymous	T→C	F8F
LD6_0250_phi1	Pf6*	ZDSLLIWT_CDS_0008	hypothetical protein	SNP	nonsynonymous	A→G	T29A
LD6_0250_phi1	Pf6*	ZDSLLIWT_CDS_0008/MPAO1_RS24215	hypothetical protein/hypothetical protein	SNP	intergenic	T→C	N/A
LD6_0250_phi1	Pf6*	MPAO1_RS24205	hypothetical protein	SNP	synonymous	A→C	P315P
LD6_0250_phi1	Pf6*	MPAO1_RS24205	hypothetical protein	SNP	synonymous	T→C	P318P
LD6_0250_phi1	Pf6*	MPAO1_RS24205	hypothetical protein	SNP	synonymous	T→C	T329T
LD6_0250_phi1	Pf6*	MPAO1_RS24205	hypothetical protein	SNP	nonsynonymous	A→C	E343D
LD6_0250_phi1	Pf6*	MPAO1_RS24205	hypothetical protein	SNP	synonymous	C→T	D357D
LD6_0250_phi1	Pf6*	MPAO1_RS24205	hypothetical protein	SNP	synonymous	T→C	G361G
LD6_0250_phi1	Pf6*	MPAO1_RS24205	hypothetical protein	SNP	synonymous	A→G	L406L
LD6_0250_phi1	Pf6*	MPAO1_RS24205	hypothetical protein	SNP	synonymous	T→C	A412A
LD6_0250_phi1	Pf6*	ZDSLLIWT_CDS_0012	minor head protein Ace-like	SNP	synonymous	T→C	D20D

LD6_0250_phi1	Pf6*	ZDSLLIWT_CDS_0012	minor head protein Ace-like	SNP	synonymous	T→C	S66S
LD6_0250_phi1	Pf6*	ZDSLLIWT_CDS_0012	minor head protein Ace-like	SNP	synonymous	G→T	G82G
LD6_0250_phi1	Pf6*	MPAO1_RS24195	CTXphi Zot-like toxin	SNP	synonymous	C→T	F49F
LD6_0250_phi1	Pf6*	MPAO1_RS24195	CTXphi Zot-like toxin	SNP	synonymous	G→T	G82G
LD6_0250_phi1	Pf6*	MPAO1_RS24195	CTXphi Zot-like toxin	SNP	synonymous	G→A	K96K
LD6_0250_phi1	Pf6*	MPAO1_RS24195	CTXphi Zot-like toxin	SNP	synonymous	A→G	A117A
LD6_0250_phi1	Pf6*	MPAO1_RS24195	CTXphi Zot-like toxin	SNP	synonymous	C→T	H131H
LD6_0250_phi1	Pf6*	MPAO1_RS24195	CTXphi Zot-like toxin	SNP	synonymous	C→T	I138I
LD6_0250_phi1	Pf6*	MPAO1_RS24195	CTXphi Zot-like toxin	SNP	synonymous	A→G	Q215Q
LD6_0250_phi1	Pf6*	MPAO1_RS24195	CTXphi Zot-like toxin	SNP	nonsynonymous	G→C	G254A
LD6_0250_phi1	Pf6*	MPAO1_RS24195	CTXphi Zot-like toxin	SNP	nonsynonymous	G→A	A281T
LD6_0250_phi1	Pf6*	MPAO1_RS24195	CTXphi Zot-like toxin	SNP	synonymous	C→T	L318L
LD6_0250_phi1	Pf6*	MPAO1_RS24195	CTXphi Zot-like toxin	SNP	synonymous	T→C	L334L
LD6_0250_phi1	Pf6*	MPAO1_RS24195	CTXphi Zot-like toxin	SNP	nonsynonymous	G→C	E335D
LD6_0250_phi1	Pf6*	MPAO1_RS24195	CTXphi Zot-like toxin	SNP	synonymous	G→A	V356V
LD6_0250_phi1	Pf6*	MPAO1_RS24195	CTXphi Zot-like toxin	SNP	nonsynonymous	C→G	D370E
LD6_0250_phi1	Pf6*	MPAO1_RS24195	CTXphi Zot-like toxin	SNP	nonsynonymous	G→A	A378T
LD6_0250_phi1	Pf6*	MPAO1_RS24195	CTXphi Zot-like toxin	SNP	nonsynonymous	G→C	A382P
LD6_0250_phi1	Pf6*	MPAO1_RS24195	CTXphi Zot-like toxin	SNP	nonsynonymous	C→T	A389V
LD6_0250_phi1	Pf6*	MPAO1_RS24195	CTXphi Zot-like toxin	SNP	synonymous	C→T	V394V
LD6_0250_phi1	Pf6*	MPAO1_RS24195	CTXphi Zot-like toxin	SNP	synonymous	G→C	A396A
LD6_0250_phi1	Pf6*	MPAO1_RS24195	CTXphi Zot-like toxin	SNP	nonsynonymous	T→C	V400A
LD6_0250_phi1	Pf6*	MPAO1_RS24195	CTXphi Zot-like toxin	SNP	synonymous	T→G	G404G
LD6_0250_phi1	Pf6*	MPAO1_RS24195	CTXphi Zot-like toxin	SNP	nonsynonymous	C→G	H423Q
LD6_0250_phi1	Pf6*	ZDSLLIWT_CDS_0014	hypothetical protein	SUB		Δ2bp	N/A
LD6_0250_phi1	Pf6*	ZDSLLIWT_CDS_0014	hypothetical protein	SNP	nonsynonymous	T→A	S21T
LD6_0250_phi1	Pf6*	ZDSLLIWT_CDS_0014/MPAO1_RS24190	hypothetical protein/hypothetical protein	SNP	intergenic	T→C	N/A
LD6_0250_phi2	Pf6*	MPAO1_RS28990/MPAO1_RS24230	transcriptional regulator/hypothetical protein	SNP	intergenic	C→T	N/A
LD6_0250_phi2	Pf6*	MPAO1_RS28990/MPAO1_RS24230	transcriptional regulator/hypothetical protein	DEL		Δ4bp	N/A
LD6_0250_phi2	Pf6*	MPAO1_RS24205	hypothetical protein	SNP	synonymous	T→C	P318P
LD6_0250_phi2	Pf6*	MPAO1_RS24205	hypothetical protein	SNP	synonymous	T→C	T329T
LD6_0250_phi2	Pf6*	MPAO1_RS24205	hypothetical protein	SNP	nonsynonymous	A→C	E343D
LD6_0250_phi2	Pf6*	MPAO1_RS24205	hypothetical protein	SNP	synonymous	C→T	D357D
LD6_0250_phi2	Pf6*	MPAO1_RS24205	hypothetical protein	SNP	synonymous	T→C	G361G
LD6_0250_phi2	Pf6*	MPAO1_RS24205	hypothetical protein	SNP	synonymous	A→G	L406L
LD6_0250_phi2	Pf6*	MPAO1_RS24205	hypothetical protein	SNP	synonymous	T→C	A412A
LD6_0250_phi2	Pf6*	ZDSLLIWT_CDS_0012	minor head protein Ace-like	SNP	synonymous	T→C	D20D
LD6_0250_phi2	Pf6*	ZDSLLIWT_CDS_0012	minor head protein Ace-like	SNP	synonymous	T→C	S66S
LD6_0250_phi2	Pf6*	ZDSLLIWT_CDS_0012	minor head protein Ace-like	SNP	synonymous	G→T	G82G
LD6_0250_phi2	Pf6*	MPAO1_RS24195	CTXphi Zot-like toxin	SNP	synonymous	C→T	F49F
LD6_0250_phi2	Pf6*	MPAO1_RS24195	CTXphi Zot-like toxin	SNP	synonymous	G→T	G82G
LD6_0250_phi2	Pf6*	MPAO1_RS24195	CTXphi Zot-like toxin	SNP	synonymous	G→A	K96K

LD6_0250_phi2	Pf6*	MPAO1_RS24195	CTXphi Zot-like toxin	SNP	synonymous	A→G	A117A
LD6_0250_phi2	Pf6*	MPAO1_RS24195	CTXphi Zot-like toxin	SNP	synonymous	C→T	H131H
LD6_0250_phi2	Pf6*	MPAO1_RS24195	CTXphi Zot-like toxin	SNP	synonymous	C→T	I138I
LD6_0250_phi2	Pf6*	MPAO1_RS24195	CTXphi Zot-like toxin	SNP	synonymous	A→G	Q215Q
LD6_0250_phi2	Pf6*	MPAO1_RS24195	CTXphi Zot-like toxin	SNP	nonsynonymous	G→C	G254A
LD6_0250_phi2	Pf6*	MPAO1_RS24195	CTXphi Zot-like toxin	SNP	nonsynonymous	G→A	A281T
LD6_0250_phi2	Pf6*	MPAO1_RS24195	CTXphi Zot-like toxin	SNP	synonymous	C→T	L318L
LD6_0250_phi2	Pf6*	MPAO1_RS24195	CTXphi Zot-like toxin	SNP	synonymous	T→C	L334L
LD6_0250_phi2	Pf6*	MPAO1_RS24195	CTXphi Zot-like toxin	SNP	nonsynonymous	G→C	E335D
LD6_0250_phi2	Pf6*	MPAO1_RS24195	CTXphi Zot-like toxin	SNP	synonymous	G→A	V356V
LD6_0250_phi2	Pf6*	MPAO1_RS24195	CTXphi Zot-like toxin	SNP	nonsynonymous	C→G	D370E
LD6_0250_phi2	Pf6*	MPAO1_RS24195	CTXphi Zot-like toxin	SNP	nonsynonymous	G→A	A378T
LD6_0250_phi2	Pf6*	MPAO1_RS24195	CTXphi Zot-like toxin	SNP	nonsynonymous	G→C	A382P
LD6_0250_phi2	Pf6*	MPAO1_RS24195	CTXphi Zot-like toxin	SNP	nonsynonymous	C→T	A389V
LD6_0250_phi2	Pf6*	MPAO1_RS24195	CTXphi Zot-like toxin	SNP	synonymous	C→T	V394V
LD6_0250_phi2	Pf6*	MPAO1_RS24195	CTXphi Zot-like toxin	SNP	synonymous	G→C	A396A
LD6_0250_phi2	Pf6*	MPAO1_RS24195	CTXphi Zot-like toxin	SNP	nonsynonymous	T→C	V400A
LD6_0250_phi2	Pf6*	MPAO1_RS24195	CTXphi Zot-like toxin	SNP	synonymous	T→G	G404G
LD6_0250_phi2	Pf6*	MPAO1_RS24195	CTXphi Zot-like toxin	SNP	nonsynonymous	C→G	H423Q
LD6_0250_phi2	Pf6*	ZDSLLIWT_CDS_0014	hypothetical protein	SUB		Δ2bp	N/A
LD6_0250_phi2	Pf6*	ZDSLLIWT_CDS_0014	hypothetical protein	SNP	nonsynonymous	T→A	S21T
LD6_0250_phi2	Pf6*	ZDSLLIWT_CDS_0014/MPAO1_RS24190	hypothetical protein/hypothetical protein	SNP	intergenic	T→C	N/A
LD6_0250_phi3	Pf6	MPAO1_RS28990/MPAO1_RS24230	transcriptional regulator/hypothetical protein	SNP	intergenic	C→T	N/A
LD6_0250_phi3	Pf6	MPAO1_RS28990/MPAO1_RS24230	transcriptional regulator/hypothetical protein	DEL		Δ4bp	N/A
LD6_0250_phi3	Pf6	MPAO1_RS24215	hypothetical protein	SNP	nonsynonymous	A→C	Q8P
LD6_0500_phi1	Pf6	MPAO1_RS28990/MPAO1_RS24230	transcriptional regulator/hypothetical protein	SNP	intergenic	C→T	N/A
LD6_0500_phi1	Pf6	MPAO1_RS28990/MPAO1_RS24230	transcriptional regulator/hypothetical protein	DEL		Δ4bp	N/A
LD6_0500_phi1	Pf6	MPAO1_RS24215	hypothetical protein	SNP	nonsynonymous	A→C	Q8P
LD6_0500_phi1	Pf6	MPAO1_RS24195	CTXphi Zot-like toxin	SNP	nonsynonymous	C→T	R355W
LD6_0500_phi2	Pf6	MPAO1_RS28990/MPAO1_RS24230	transcriptional regulator/hypothetical protein	SNP	intergenic	C→T	N/A
LD6_0500_phi2	Pf6	MPAO1_RS28990/MPAO1_RS24230	transcriptional regulator/hypothetical protein	DEL		Δ4bp	N/A
LD6_0500_phi2	Pf6	MPAO1_RS24215	hypothetical protein	SNP	nonsynonymous	A→C	Q8P
LD6_0500_phi3	Pf6	MPAO1_RS28990/MPAO1_RS24230	transcriptional regulator/hypothetical protein	SNP	intergenic	C→T	N/A
LD6_0500_phi3	Pf6	MPAO1_RS28990/MPAO1_RS24230	transcriptional regulator/hypothetical protein	DEL		Δ4bp	N/A
LD6_0500_phi3	Pf6	MPAO1_RS24215	hypothetical protein	SNP	nonsynonymous	A→C	Q8P
LD8_0250_phi1	Pf4	[PA0716.1]	[PA0716.1]	DEL		Δ141bp	N/A

LD8_0250_phi2	Pf4	[PA0716.1]	[PA0716.1]	DEL		Δ141bp	N/A
LD8_0500_phi1	Pf4	[PA0716.1]	[PA0716.1]	DEL		Δ141bp	N/A
LD8_0500_phi1	Pf4	PA0720	single strand DNA binding protein	SNP	nonsynonymous	C→TRUE	P82S
LD8_0500_phi2	Pf4	[PA0716.1]	[PA0716.1]	DEL		Δ141bp	N/A
LD8_0500_phi3	Pf4	[PA0716.1]	[PA0716.1]	DEL		Δ141bp	N/A
LD8_0500_phi3	Pf4	PA0720	single strand DNA binding protein	SNP	nonsynonymous	C→TRUE	P82S

*indicates recombinant phage isolates.
